# Phage therapy for severe bacterial infections: a narrative review

**DOI:** 10.5694/mja2.50355

**Published:** 2019-10-06

**Authors:** Aleksandra Petrovic Fabijan, Ali Khalid, Susan Maddocks, Josephine Ho, Timothy Gilbey, Indy Sandaradura, Ruby CY Lin, Nouri Ben Zakour, Carola Venturini, Bethany Bowring, Jonathan R Iredell

**Affiliations:** ^1^ Centre for Infectious Diseases and Microbiology Westmead Hospital Sydney NSW; ^2^ Westmead Institute for Medical Research Sydney NSW; ^3^ University of Sydney Sydney NSW; ^4^ Westmead Hospital Sydney NSW; ^5^ Wagga Wagga Base Hospital Wagga Wagga NSW

**Keywords:** Bacterial infections, Infection control

## Abstract

- Bacteriophage (phage) therapy is re‐emerging a century after it began.
- Activity against antibiotic‐resistant pathogens and a lack of serious side effects make phage therapy an attractive treatment option in refractory bacterial infections.
- Phages are highly specific for their bacterial targets, but the relationship between in vitro activity and in vivo efficacy remains to be rigorously evaluated.
- Pharmacokinetic and pharmacodynamic principles of phage therapy are generally based on the classic predator–prey relationship, but numerous other factors contribute to phage clearance and optimal dosing strategies remain unclear.
- Combinations of fully characterised, exclusively lytic phages prepared under good manufacturing practice are limited in their availability.
- Safety has been demonstrated but randomised controlled trials are needed to evaluate efficacy.

The bactericidal properties of the waters of the Ganges and the Jumna were first described at the end of the 19th century by British bacteriologist Ernest Hankin.^1^ Countryman and fellow microbiologist Frederick Twort's subsequent studies^2^ were interrupted by the First World War, but he is often credited as the discoverer of what was later termed the “Twort–d'Herelle phenomenon”.^3^ It was the French Canadian Felix d'Herelle who first proposed the term “bacteriophage” (phage) (ie, bacterium eater)^4^ and pioneered its therapeutic application using oral concentrates to treat *Shigella* enteritis (bacillary dysentery) in 1919, in the first recorded description of phage therapy. In the years that followed, phages were used with varying success by d'Herelle and others for a variety of serious infections, including staphylococcal bacteraemia, typhoid fever and osteomyelitis,^5^ and d'Herelle was awarded the Leeuwenhoek Medal in 1925 for his contributions to applied microbiology. Bacteriophages were visualised for the first time in 1939, when electron microscopy was developed, and a morphotypic classification was subsequently proposed by Helmut Ruska in 1943.^6^ Such classifications are not well aligned with genetic relatedness^7^ and a detailed discussion is beyond the scope of this review, but for our purposes, the most commonly used therapeutic phages are the double‐stranded DNA viruses (often ~ 100–200 kbp) that are grouped together as “tailed viruses” (order *Caudovirales*) (Box 1). Phages are now understood to be the most diverse and abundant life form on Earth, probably outnumbering bacteria by ten to one.^8^

Box 1 **Bacteriophage morphology**

**Figure:**
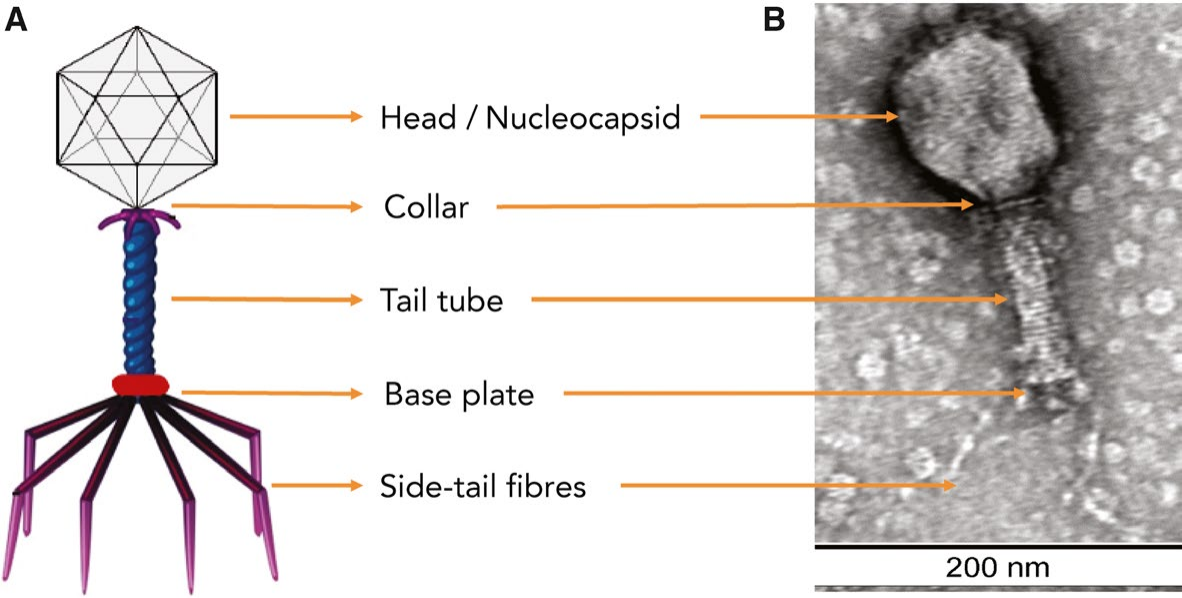
Schematic (**A**) and electron micrograph of bacteriophage (*Myoviridae*) with 1% uranyl acetate negative staining, with size marker (**B**). Bacteriophage preparations were dialysed against 0.1 M ammonium acetate in dialysis cassettes with a 10 000 membrane molecular weight cut‐off (Pierce Biotechnology), negatively stained with 2% uranyl acetate, and visualised using transmission electron microscopy (TEM). TEM was conducted at the Westmead Scientific Platforms (Westmead Hospital, Sydney, Australia) on a Philips CM120 BioTWIN (Thermo Fisher Scientific) transmission electron microscope at 100 kV. Images were recorded with a SIS Morada digital camera using iTEM software (Olympus Soft Imaging Solutions).

Antibiotic resistance is widely recognised as a fundamental threat to human health, development and security,^9^ but new antimicrobial drug development is largely unprofitable. Therefore, it is unsurprising that phage therapy is under scrutiny again. Phages have antimicrobial efficacy that correlates with their in vitro activity^10^ and are generally regarded as safe. The first review of intravenous phage for sepsis in 1931 described hundreds of successes,^5^ but enthusiasm waned as antibiotics arrived, especially in booming post‐war capitalist economies, remaining popular only in the Eastern bloc, where it was well supported.^11^

Clinical implementation via current drug development and clinical practice guidelines is problematic.^12^, ^13^ Much of the older literature suffered from experimental and technical problems,^14^ and most clinicians believe that phage therapy has “not yet been [rigorously] investigated”.^15^ However, after a century of use, there is increasing demand for rigorous evaluation of phage therapy, including in the most severe infections.^16^ There are case series with generally good outcomes, particularly in osteoarticular infections,^17^ diabetic foot infections,^18^ and chronic prostatitis.^19^, ^20^, ^21^ Two reviews^22^, ^23^ detail the history of phage therapy in Russia and Eastern European countries and its application in specific human infections, a 2012 article^24^ provides a historical review of phage therapy over the years, and comprehensive up‐to‐date reviews are now also available.^25^

However, randomised controlled trial (RCT) experience in humans is less impressive (Box 2). The largest reported RCT was conducted in Russia in 1963–64.^23^ Tens of thousands of children received either anti‐*Shigella* phage or placebo. The incidence of persisting clinical and culture‐confirmed *Shigella* dysentery was 3.8‐fold and 2.6‐fold higher, respectively, in the placebo group. In contrast, a case–control trial (*n *= 8) in the 1970s of high dose phage for the treatment of cholera found tetracyclines to be more effective.^26^ A recent RCT of a well characterised phage cocktail demonstrated safety but not efficacy for the treatment of diarrhoeal illnesses in Bangladeshi children,^27^, ^29^ but only 60% of enrolled patients had proven *Escherichia coli* infection, and only half of these were phage‐susceptible in vitro. More recently, a phase 1 double‐blind RCT of a topical bacteriophage cocktail in chronic venous leg ulcers found no safety concerns,^30^ and the much‐anticipated PhagoBurn trial (phase 1/2 RCT),^10^ comparing topical application of a cocktail of phages against *Pseudomonas* with standard care with silver sulfadiazine, found that significant reductions in bacterial counts took longer in the phage group and that the silver sulfadiazine group had higher treatment success than the phage group. The PhagoBurn trial suffered from instability of the phage preparation, but showed that phage‐susceptibility of *Pseudomonas* isolates in vitro is crucial to eventual clinical success, with susceptibility rates of 89% in successful cases compared with 24% in clinical failures in the phage therapy arm (Box 2).

Box 2 **Randomised controlled trials on phage therapy**

**Table:** 

Year	Number of participants	Study design and follow‐up	Intervention and duration of therapy	Main outcome	Comments
1963–64	30 769	Prospective placebo‐controlled RCT of *Shigella* dysentery; follow‐up 109 days^24^	Phage preparation peroral once a week; therapy 109 days	Incidence of dysentery 3.8 times higher in placebo group Microbiologically confirmed *Shigella* dysentery 2.6 times higher in placebo group	Effectiveness reported to be greater in infants aged 6–12 months and lowest in children aged 5–7 years Original article in Russian, information drawn from review
1970	8	Case–control study of *Vibrio cholerae*; no follow‐up^26^	High dose of phage (10^13^ PFU), half‐hourly to hourly, until diarrhoea resolved; therapy 5–6 days	Duration and volume diarrhoea reduced in 4/8 patients; tetracycline more effective *V. cholerae* excretion cleared within 18 hours in responders	Loss of *V. cholerae* motility reported within 90 minutes of dosing No phage resistance reported after therapy; no statistical analyses reported
2009	39	Prospective double‐blind RCT of chronic venous leg ulcers; no follow‐up^27^	Phage application via ultrasonic debridement device, followed by wound dressing and bandage; therapy 12 weeks	Safety demonstrated: no significant adverse events Ulcer healing 12 weeks and 24 weeks — no difference (not powered for this)	Bacteriophage‐impregnated dressings included lactoferrin
2009	24	Prospective double‐blind RCT of chronic otitis externa; *Pseudomonas aeruginosa;* follow‐up 6 weeks^28^	10^5^ PFU phage application plus meticulous ear cleaning; single application	Significant improvement in symptoms and signs of otitis in phage‐treated group, and in erythema for phage *v* control at Day 7 (*P *= 0.02) and Day 21 (*P *= 0.014); ulceration, granulation and polyps at Day 7 (*P *= 0.017) and Day 42 (*P *= 0.025) 3/12 phage‐treated patients demonstrated near‐complete reduction in all visual analogue scores, along with undetectable *P. aeruginosa*, compared with none in the placebo group	Trial stopped early during interim analysis by primary investigator due to clinical improvement in the phage‐treated group Phage replication in vivo was detected for a mean of 23.1 days (median, 21 days) in the treatment group
2016	120	Prospective double‐blind RCT of acute diarrhoea in children; follow‐up 21 days^29^	T4 phage cocktail or Microgen ColiProteus phage cocktail; therapy 4 days	No difference in stool load or frequency between groups Safety: no adverse events No significant phage replication in stool (stool output < oral input). No difference in phage titres seen between stool with phage‐sensitive *Escherichia coli* compared with those without phage‐sensitive *E. coli*	Trial stopped early at interim analysis due to perceived lack of efficacy Phage interventions targeted *E. coli* but only 60% of participants had confirmed pathogenic *E. coli* Diarrhoeal illnesses were probably the result of complex polymicrobial interplay — potential explanation for a lack of efficacy of narrow spectrum phage treatment
2019	27	Prospective double‐blind RCT of clinically infected burn wounds (*P. aeruginosa*); follow‐up 14 days^10^	Alginate template soaked in PP1131 at ~ 1 × 10^6^ PFU/mL, applied topically to wounds daily; therapy 7 days	Time to sustained reduction in two‐quadrant bacterial burden was significantly longer in the phage group (median, 144 h [95% CI, 48–NR] *v* 47 h [95% CI, 23–122]; HR, 0.29 [95% CI, 0.10–0.79]; *P* = 0.018) Higher proportion of participants had successful treatment outcome in the standard care group than in the phage group (13/17 [76%] *v* 9/17 [53%]; *P *= 0.15)	Study population heterogeneity: patients in the phage group were older whereas patients in the standard care group had more severe burns The actual phage dose delivered to patients was substantially lower than anticipated There were phage‐susceptibility differences in phage‐treated patients who had clinical success (89% susceptible) *v* those that failed (24% susceptible)

HR = hazard ratio; NR = not reached; PFU = plaque‐forming unit; PP1131 = cocktail of 12 natural lytic anti‐*P. aeruginosa* bacteriophages; RCT = randomised controlled trial.

## Determining bacterial susceptibility to phage infection

Phages usually exhibit a high degree of host specificity and are most easily sourced from the habitat of their usual bacterial hosts. Phages specific for clinically relevant bacteria may be readily sourced from human and animal sources, hospital wastewater, and environmental soil and water. Their reproduction is dependent on the bacterial host, either integrated into the bacterial genome as a prophage or acting in a purely parasitic manner (Box 3), the latter characteristic being exploited in phage therapy.

Box 3 **Bacteriophage life cycles**

**Figure:**
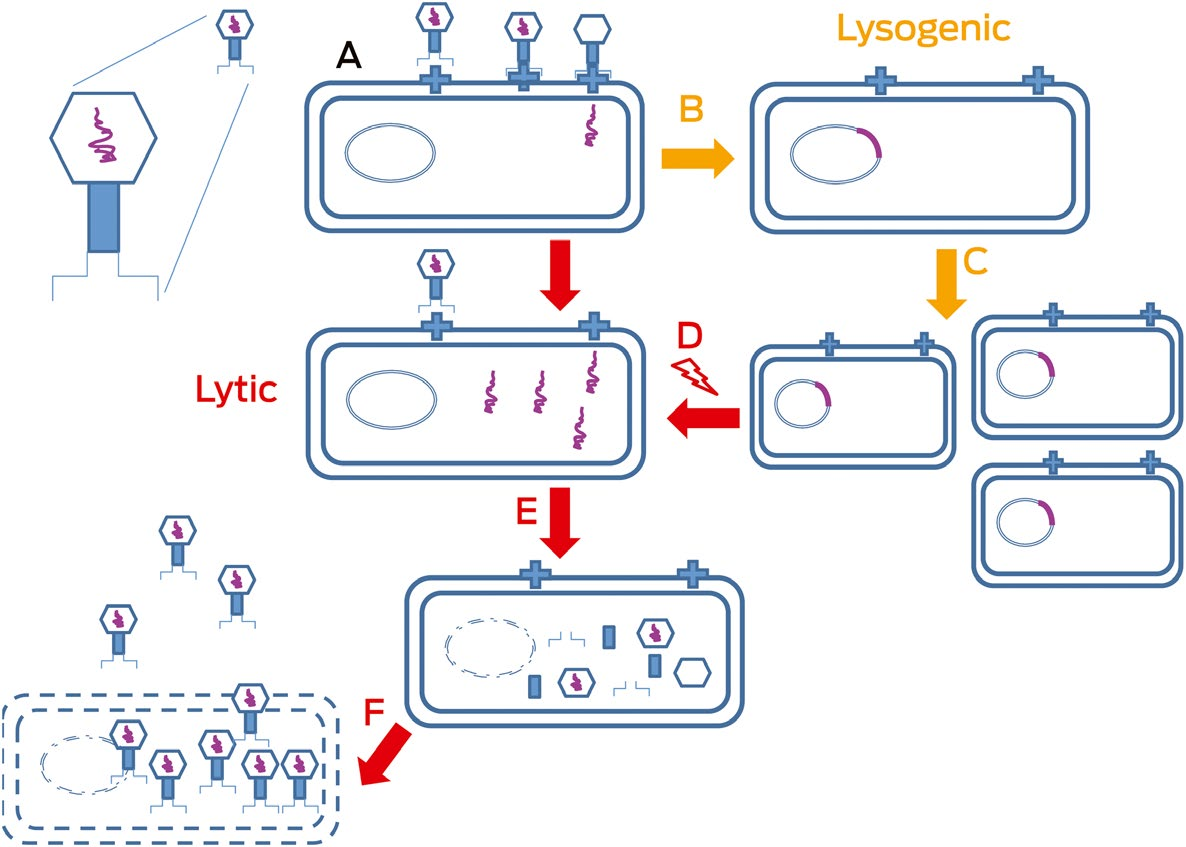
After infection (**A**), the phage DNA (purple) is classically either reproduced and packaged as new virions at the expense of the cell (a virulent phage in a lytic cycle; left, **A**,** E** and **F**) or reproduces with the host DNA (a temperate phage in a lysogenic cycle; right, **B**,** C** and **D**).

Phages bind specific receptors and adsorb onto bacterial surfaces before injecting DNA to start viral, and alter bacterial, processes (Box 3, A); their relationships range from parasitic to mutualistic.^31^, ^32^ Some integrate into bacterial chromosomes (Box 3, B) and reproduce normally (Box 3, C); lytic infection occurs (eg, when the host is stressed) (Box 3, D), then production and liberation of new virions occur (Box 3, E): lysogenised bacteria with integrated prophage (Box 3, D, red line) are protected from superinfecting phages of the same type. However, surface receptor variation, blocking of phage DNA injection, restriction or modification systems, and adaptive immunity (CRISPRs) are also protective. The ideal therapeutic phage is probably exclusively parasitic (it is virulent in the lytic cycle) and does not enter into the other common phage lifestyle, in which it integrates into and replicates with the host bacterial genome (ie, is temperate in the lysogenic cycle — a temperate phage results in a lysogenic state in the bacterial host because it may cause lysis) (Box 3, F, red arrow).

The conventional method of measuring the lytic activity of bacteriophages is by mixing target bacteria into soft agar overlaid onto a standard nutritious agar surface.^33^ Tenfold serial dilutions of candidate phage are then spotted onto the soft agar. The development of plaques after overnight incubation indicates productive phage infection and bacterial lysis to release progeny (Box 4, A). The productivity of a given infection (eg, as estimated in a plaque assay) is often referred to as the “burst size”, and the relationship between the inoculum required to generate productive infection in the original propagating host in vitro and the target organism (intended prey) is referred to as the “efficiency of plating”.

Box 4 **Bacteriophage susceptibility testing**

**Figure:**
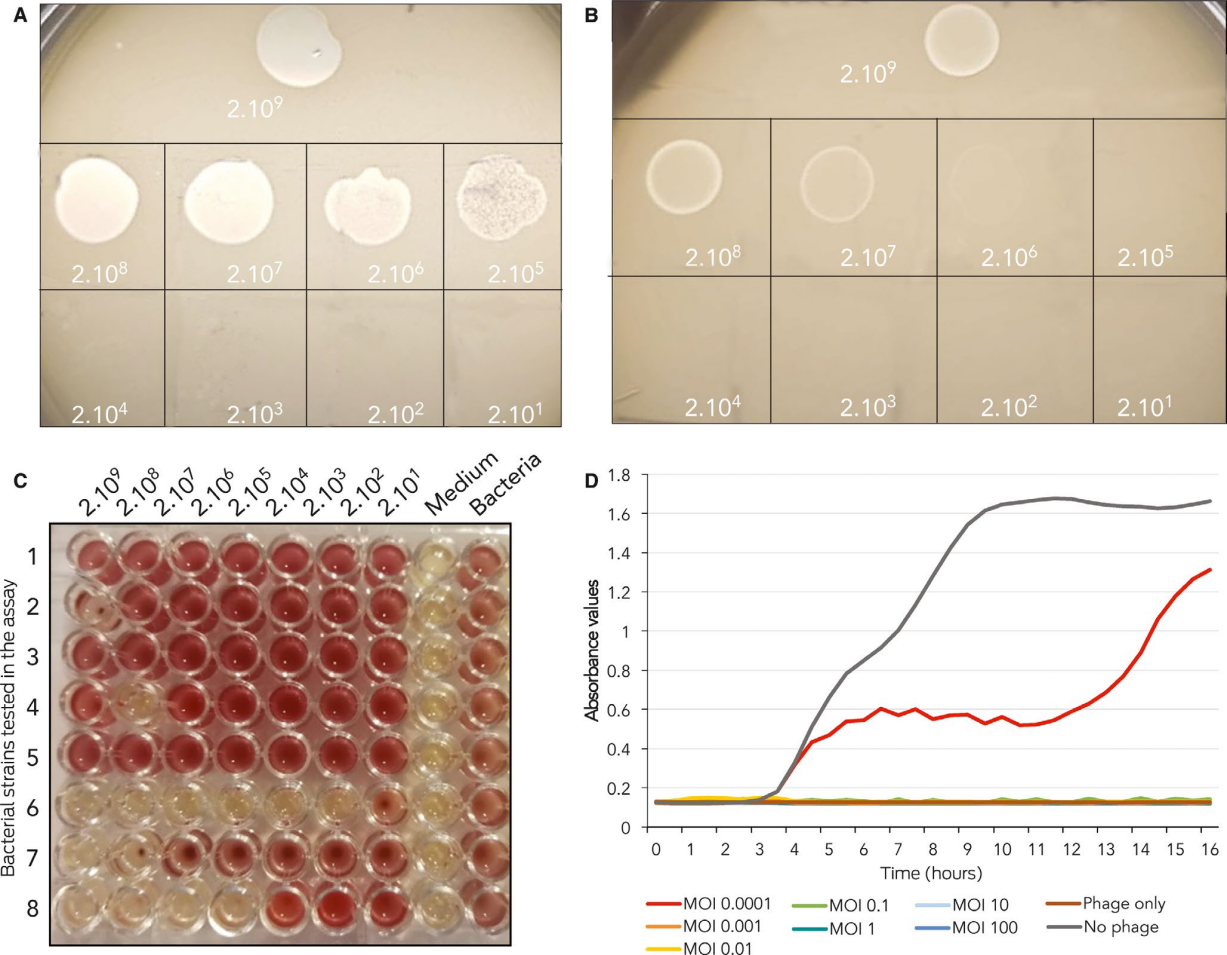
Phage‐susceptibility testing of bacterial isolates with the standard double‐layer method:^33^ (**A**) susceptible isolate — productive infection and formation of individual plaques detectable at serially diluted phage lysate — and (**B**) resistant isolate — single plaques absent and early bacterial lysis present only at high concertation of phages. Liquid culture‐based system: (**C**) across a gradient of multiplicity of infections (MOIs) against eight bacteria on a single 96‐well plate. There are seven MOIs tested from highest to lowest (left to right). Column eight and nine are phage‐only and bacteria‐only controls. (**D**) Bacterial growth kinetics in presence of phage at differing MOIs.

The plaque assay is time‐consuming and operator‐dependent, with automated methods still in their infancy. Liquid culture‐based systems that continuously monitor bacterial growth in the presence of bacteriophage in a standard 96‐well plate format (Box 4, C) may be used to infer bacterial susceptibility to phage and also to study phage–antibiotic synergy. The relationship between various in vitro assays and in vivo outcomes is poorly validated, but most microbiologists would intuit that lack of therapeutic efficacy is predictable if the phage is inactive against the target pathogen in vitro, and there are supportive data from at least one RCT in humans.^10^ A failure to produce any plaques at all in a bacterial lawn is generally regarded as a negative result (resistant bacteria) in the standard double‐layer method (Box 4, B)

The development of in vitro bacterial resistance was a prominent feature of phage therapy for a high burden multidrug‐resistant *Acinetobacter* infection when antibiotics were failing.^34^ However, resistance was not observed in a small cohort of people with severe staphylococcal infection treated intravenously with a good manufacturing practice (GMP)‐quality combination of phages^35^ when used in conjunction with effective antibiotics.^36^ The antibiotic–phage synergy issue remains complex and more work needs to be done to understand the best way to use them together.

## Phage dosing and kinetics: pharmacokinetics, pharmacodynamics and multiplicity of infection

A good understanding of the non‐linear kinetics of bacteriophage distribution in blood and tissues is necessary to maximise efficacy of future therapeutic protocols.^37^, ^38^ Usually applied to ecological rather than pharmacological systems, the phage replication cycle is generally held to follow classic Lotka–Volterra dynamics of predator (phage) and prey (bacteria), based on population sizes and interactions between them.^39^ Classic pharmacokinetic principles, predator–prey, infectious disease model dynamics, and host immune responses must all be considered, but phage pharmacokinetics (phage distribution and clearance), pharmacodynamics (predator–prey dynamics), and ratio of phages to bacteria (multiplicity of infection [MOI], perhaps better described in terms of initial MOI_input_)^40^, ^41^ are subject to many, often poorly defined, variables that may influence outcomes.^42^

Numerous methods of bacteriophage delivery have been explored, including topical, inhalational, oral and injectable (intravenous, intramuscular, subcutaneous and direct intralesional). When administering phages orally, concern has been raised regarding recombination between modular phage genomes in the gut, although there has been little evidence from trials to date to resolve this one way or the other.^43^ Phages generally have poor oral bioavailability,^16^ but intravenous delivery is efficient to virtually all organs and tissues^44^ and first‐dose kinetics can be modelled to some extent by standard techniques,^45^ especially after initial parenteral dosing. Original observations,^5^ recently repeated overseas^34^ and in Australian studies of severe sepsis,^36^ show that intravenous phage is cleared typically in the first 60 minutes. However, phage clearance is also enhanced by the mammalian innate immune responses to infection^46^ — this may affect (or be affected by) amplification and/or phage dosage (the ratio of phages to bacteria; ie, the MOI). An MOI below 0.1 is effective in mouse models,^47^ but an optimal MOI to use in humans has been suggested to be ten or over.^48^ Bacterial concentrations are 10^1^–10^5^ (more usually < 10^3^) colony‐forming units per mL of blood in severe sepsis,^49^ and thus a dose of 10^9^ plaque‐forming units (PFU) into the human blood volume (~ 5 L) is expected to yield an MOI_input_ over 200.

The paradoxical persistence of a narrow host range for phages is not fully understood, given the presumed ecological advantages^50^ of host switching when starved for prey, but phages tend to disappear as their food supply is exhausted. Thus, while clearance after intravenous dosing is primarily by the reticuloendothelial system, liver and spleen, phage populations may follow more classic predator–prey kinetics when directly administered, even in areas such as the bladder.^51^ There are well recognised limitations to the efficacy of phages in mixed bacterial infections, and infections with low bacterial density.^52^, ^53^ Therefore, in theory, antibiotics that dramatically reduce the density of target bacteria may result in phage clearance rates that exceed propagation rates and thereby limit this unique property of phages as antimicrobials,^54^ adding further to the complexities of phage kinetics.

Urine concentration of bacteriophage may be dose‐dependant,^55^, ^56^ and renal clearance of bacteriophages does not appear to be of the same magnitude as clearance by the liver and spleen.^46^ The biofilm also presents a special case. Experimental data suggest that pre‐treatment of biofilm infections with antibiotics enhances synergistic killing by phages,^57^ but biofilms can be an impenetrable layer and some bacteria such as *E. coli* have added additional barriers (curli fibres) as a collective protection.^58^

## Selection of phages for therapy

Optimised preparation of phage combinations (cocktails) for use in humans requires well characterised bacteriophages, a good understanding of the bacterial target population, and highly effective purification protocols to avoid inflammatory responses to contaminating residual bacterial endotoxin and protein.^12^, ^59^, ^60^ Toxins, resistance genes, virulence determinants, and capacity to integrate (temperate phage) are excluded by sequencing.^43^, ^61^ In vitro susceptibility testing remains specialised and should be conducted by an accepted method in a laboratory that can demonstrate reliable reproducibility of results.

General recommendations regarding the standardisation of key components of phage therapy have been published,^62^ and most authorities suggest that therapeutic mixtures include three to five phages at high titre (10^9^–10^10^ PFU/mL) with unique but overlapping host ranges to guarantee lysis of the bacterial target and clinically relevant variants.^52^, ^63^ Each phage component should ideally target different receptors to reduce the likelihood of phage‐resistant mutants arising.^37^ Phage stocks should be routinely monitored for viability and concentration, and may require ad hoc modification as target bacterial populations change over time.^12^, ^60^

Phage therapy may be presented as a discrete quality‐assured formulation either as several phages admixed to provide a broad spectrum of activity in a single medicine for a specific infection (eg, common staphylococci^35^ or infecting pathogens in a burn wound),^10^ or even by engineering additional or altered breadth of spectrum into a single optimised phage.^64^ This concept is similar to the traditional pharmaceutical model presented to regulators such as the Food and Drug Administration in the United States.

At the other extreme, specific linking of a virulent phage to the target pathogen might be done by using a large library of stable high quality preparations that can be matched quickly and efficiently in a standardised susceptibility testing format. Chosen phages can then be prepared for co‐administration by trained staff, like any other medicine, perhaps in a compounding pharmacy such as was once common in most hospitals. This latter concept is gaining prominence in Europe as a pathway to regulation, progressing particularly in Belgium and France, as the “magistral phage” approach.^65^, ^66^

## Lessons from recent Australian experience

While phages have many of the characteristics of ideal personalised medicines, lack of double‐blind phase 3 (efficacy) clinical trials in humans means that phage therapy still has the status of an old technology based on anecdotal evidence, not much different to traditional medicines. Nevertheless, there are some successful phase 1 and 2 studies, including in Australia, which suggest the potential of phage therapy as an alternative or adjunct to antibiotics.^16^, ^24^

Recent studies in Australian centres of both intranasal instillation^67^ and intravenous injection^68^ of a high purity preparation of antistaphylococcal phages^36^ have demonstrated safety and tolerability, but these preparations are not yet generally available. In the largest single uncontrolled, interventional clinical cohort study at Westmead Hospital (Australia), 14 patients with severe *S. aureus* sepsis and infective endocarditis were treated twice daily for 2 weeks with intravenous AB‐SA01 (Armata Pharmaceuticals) — a GMP‐quality cocktail product with three bacteriophages — as adjunct to standard care.^68^, ^69^ While this study is uncontrolled, phage therapy was associated with (or at least did not prevent) reduction in bacterial burden and inflammatory response and was well tolerated with no attributed adverse reactions.^36^

Natural bacteriophages are defined as investigational drugs by the Therapeutic Goods Administration in Australia. The regulatory side of bacteriophage therapy is beyond the scope of this review, but most medical communities are both sceptical and curious.

## Conclusion

To progress, we need at least to guarantee the availability of efficient phage susceptibility testing and of preparations that are safe for intravenous administration, define optimal dosing, and effectively monitor phage–bacteria–human host interactions and any important collateral effects on other members of the microflora.

Realistically, application of therapeutic phages alone may never completely replace chemical antimicrobials as the standard of care. For now, it is reasonable to define phage therapy as a promising rescue therapy that has been associated with some spectacular results^34^, ^64^, ^70^ and is considered an increasingly essential tool with which to manage rising antimicrobial resistance, whether as multiphage cocktail preparations or as individual phages matched to specific pathogens. It is clear, however, that RCTs are needed to define phage efficacy and to deal with the issues we outline in this review before phages find their way into the general pharmacopoeia.

## Competing interests

No relevant disclosures.

## Provenance

Not commissioned; externally peer reviewed.
